# Circ-FBXW12 aggravates the development of diabetic nephropathy by binding to miR-31-5p to induce LIN28B

**DOI:** 10.1186/s13098-021-00757-x

**Published:** 2021-12-04

**Authors:** Aidong Sun, Ningshuang Sun, Xiao Liang, Zhenbo Hou

**Affiliations:** 1 Department of Endocrinology, Zibo First Hospital, Zibo, 255200 Shandong China; 2 Chinese Traditional College of Changchun University of Chinese Medicine, Changchun, 130022 Jilin China; 3 Department of Thoracic Surgery, Zibo Central Hospital, Zibo, 255000 Shandong People’s Republic of China; 4 Department of Pathology, Zibo Central Hospital, No. 54 Gongqingtuan West Road, Zhangdian District, Zibo, 255000 Shandong People’s Republic of China

**Keywords:** DN, HMCs, Circ-FBXW12, miR-31-5p, LIN28B

## Abstract

**Background:**

The involvement of circular RNAs (circRNAs) in diabetic nephropathy (DN) has been gradually identified. In this study, we aimed to explore the functions of circRNA F-box/WD repeat-containing protein 12 (circ-FBXW12) in DN development.

**Methods:**

Reverse transcription quantitative polymerase chain reaction (RT-qPCR) assay was performed for the levels of circ-FBXW12, FBXW12 mRNA, microRNA-31-5p (miR-31-5p) and Lin-28 homolog B (LIN28B) mRNA. RNase R assay was used to analyze the stability of circ-FBXW12. Cell Counting Kit-8 (CCK-8) assay, flow cytometry analysis and 5-ethynyl-2′- deoxyuridine (EdU) assay were employed to evaluate cell viability, cell cycle and proliferation, respectively. Enzyme linked immunosorbent assay (ELISA) was done to measure the concentrations of inflammatory cytokines. Western blot assay was conducted for protein levels. Superoxide dismutase (SOD) activity and malondialdehyde (MDA) level were examined with commercial kits. Dual-luciferase reporter assay and RNA immunoprecipitation (RIP) assay were performed to verify the relationships among circ-FBXW12, miR-31-5p and LIN28B.

**Results:**

Circ-FBXW12 level was increased in DN patients’ serums and high glucose (HG)-induced human mesangial cells (HMCs). Circ-FBXW12 knockdown suppressed cell proliferation, arrested cell cycle, reduced extracellular matrix (ECM) production and oxidative stress in HG-induced HMCs. Circ-FBXW12 was identified as the sponge for miR-31-5p, which then directly targeted LIN28B. MiR-31-5p inhibition reversed circ-FBXW12 knockdown-mediated effects on cell proliferation, cell cycle process, ECM production and oxidative in HG-triggered HMCs. Moreover, miR-31-5p overexpression showed similar results with circ-FBXW12 knockdown in HG-stimulated HMC progression, while LIN28B elevation reversed the effects.

**Conclusion:**

Circ-FBXW12 knockdown suppressed HG-induced HMC growth, inflammation, ECM accumulation and oxidative stress by regulating miR-31-5p/LIN28B axis.

## Introduction

Diabetes mellitus is a common metabolic disease that can cause chronic renal impairment and lead to diabetic nephropathy (DN) [1, 2]. At present, DN has been one of the major reasons for end-stage renal disease around the world [3]. The clinical features of DN include mesangial cell (MC) hyperplasia, proteinuria, extracellular matrix (ECM) accumulation, and renal fibrosis [4, 5]. Oxidative stress and inflammation caused by elevated blood glucose are considered to be inseparable factors in the occurrence of DN [6]. Hence, it is crucial to explore the mechanism of MC injury under high glucose (HG) for understanding DN development.

Circular RNAs (circRNAs) are endogenous non-coding RNAs (ncRNAs) that possess continuous covalent closed loops [7]. Mounting evidence has demonstrated that circRNAs function as vital masters in a variety of diseases by elevating mRNA expression through acting microRNA (miRNA) sponges [8]. Some circRNAs have been implied to be dysregulated and play essential roles in DN pathogenesis. For example, circ_0080425 was upregulated in DN mice model and HG-treated MCs, and promoted MC cell growth and fibrosis via circ_0080425/miR-24-3p/FGF11 regulatory axis [9]. Circ_0000285 aggravated podocyte proliferation and facilitated apoptosis in DN depending on miR-654-3p/Mitogen-Activated Protein Kinase 6 (MAPK6) axis [10]. Circ_LARP4 hampered cell proliferation and fibrosis and accelerated apoptosis in HG-induced mouse mesangial cells (MMCs) by decoying miR-424 [11]. For circRNA F-box/WD repeat-containing protein 12 (circ-FBXW12, also termed as hsa_circ_0123996), Wang et al. declared that circ_0123996 knockdown suppressed MMC proliferation and fibrosis by upregulating BTB Domain And CNC Homolog 1 (Bach1) via sponging miR-149-5p [12]. Even so, the underlying mechanism of circ-FBXW12 in DN development are poorly characterized.

The small ncRNAs, miRNAs have been demonstrated to be implicated in DN pathogenesis [13]. For example, miR-325-3p restrained renal inflammatory response and fibrosis by interacting with C–C motif chemokine ligand 19 (CCL19) [14]. MiR-15b-5p alleviated HG-induced inflammatory damage and oxidative damage in podocytes by binding to semaphorin 3A (Sema3A) [15]. Moreover, miR-31 level was declined in T2D patients with DN and negatively related to the secretion of inflammatory factors [16].

Lin-28 homolog B (LIN28B) is related to multiple human diseases, such as hepatocellular carcinoma [17], neuroendocrine prostate cancer [18], neuroblastoma [19] and Ewing sarcoma [20]. Moreover, LIN28B/let-7 was able to alter ECM production in TGF-β-induced MMCs [21]. The reports suggested that LIN28B played a vital function in DN.

With the assistance of bioinformatics tools circinteractome and starBase V2.0, miR-31-5p was found to contain the binding sequences of circ-FBXW12 and LIN28B, thus, we explored their functions and relationships in regulating DN development.

## Materials and methods

### Clinical sample acquisition

A total of 23 healthy volunteers who underwent routine health checks (Normal group), 23 type-2 diabetes patients with DN (DN group) and 14 diabetic patients without DN (DM group) at Zibo First Hospital were enrolled in the study. The diabetic patients were diagnosed according to urinary albumin excretion and divided into two groups: diabetes with normoalbuminuria (DM group) (urinary albumin excretion rate (UAER) < 30 mg/24 h and serum creatinine (Scr) < 133 μmol/L), diabetes with albuminuria (DN group) (UAER > 30 mg/24 h). The DN patients were then subdivided into two groups: microalbuminuria group (30 mg/24 h < UAER < 300 mg/24 h and macroalbuminuria group (UAER > 300 mg/24 h). The patients were excluded this study if they had a history of cardiovascular disease, morbified obesity, organic or inflammatory disease, infectious, autoimmune, hematologic disease, malignancy, fever and diabetic neuropathy. The blood samples were acquired after the research was approved by the Ethics Committee of Zibo First Hospital and written informed consents were provided by the participants. The serums were acquired through centrifugation. The clinical characteristics of the participants were exhibited in Table 1.

**Table 1:** Clinical characteristics

Parameters	Normal group (n = 23)	DN group (n = 23)
Gender (male/female)	14/9	12/11
Age (years)	55.3 ± 5.8	57.2 ± 6.5
BMI	22.6 ± 1.6	24.8 ± 2.4
Duration of diabetes (years)		7.9 ± 1.4
Fasting plasma glucose (mmol/L)	4.1 ± 0.9	7.5 ± 1.9
Blood urea nitrogen (mmol/L)	3.9 ± 1.2	7.8 ± 2.8
HbA1c (mmol/mol)	74.5 ± 10.5	40.1 ± 9.3
Serum creatinine (µmol/L)	65.3 ± 7.6	143.6 ± 39.2
Retinopathy	0	11

### Cell culture

HMCs were acquired from Procell (Wuhan, China) and cultured in Dulbecco’s modified Eagle’s medium (DMEM; Procell) plus 10% fetal bovine serum (FBS; Procell) and 1% Penicillin–Streptomycin (Procell) at 37 °C in a humid incubator consisting of 5% CO_2_. The HMCs were treated with 5.5 mM glucose (Sigma-Aldrich, St. Louis, MO, USA) for the control group or 30 mM glucose (Sigma-Aldrich) for the HG group.

### Cell transfection

To knock down circ-FBXW12 or LIN28B, small interfering RNA (siRNA) target circ-FBXW12 (si-circ-FBXW12) or LIN28B (si-LLIN28B) was transfected into HMCs with scramble control si-NC as a control. The mimics of miR-31-5p (miR-31-5p) and inhibitors of miR-31-5p (anti-miR-31-5p) were synthesized to elevate or reduce miR-31-5p expression with miR-NC or anti-miR-NC as the control. To upregulate LIN28B expression, the overexpression plasmid of LIN28B was introduced into HMCs with pcDNA as a control. All these compositions were bought from GenePharma (Shanghai, China) and cell transfection was manipulated utilizing Lipofectamine 2000 (Invitrogen, Carlsbad, CA, USA).

### Reverse transcription quantitative polymerase chain reaction (RT-qPCR) assay

The RNA was obtained utilizing TRIzol (Invitrogen) and cDNAs was generated via All-in-One™ miRNA First-Strand cDNA Synthesis Kit (GeneCopoeia, Rockville, MD, USA) or PrimeScript™ RT reagent Kit (Takara, Dalian, China) according to the manufacturers’ instructions. Then RT-qPCR was done via SYBR Premix DimerEraser (Takara). The thermocycling conditions were as follows: (1) 95 °C for 5 min; (2) 40 cycles at 95 °C for 30 s, 60 °C for 45 s and 72 °C for 30 s; and (3) dissolving curve at 94 °C for 90 s, 60 °C for 180 s and 94 °C for 10 s. The primers were exhibited in Table 2. The abundance was estimated with the 2^−ΔΔCt^ method. Glyceraldehyde 3-phosphate dehydrogenase (GAPDH) was used for the internal reference for circ-FBXW12, FBXW12 and LIN28B, while U6 was used for the internal reference for miR-31-5p.

**Table 2:** Primers sequences used for qRT-PCR

Primers	Sequences (5′–3′)	Tm	PCR product	Ct range
circ-FBXW12-forward	ACACGTGGCATGATCACACA	60.25	125	0.8
circ-FBXW12-reverse	ACTCCTGGACTGGGCTTGAC	58.4		
FBXW12-forward	CTGCCTGGGTTAAGAGATGTTT	58.31	115	1.2
FBXW12-reverse	AGGTACGACTGTATGTCCCAC	58.9		
miR-31-5p-forward	ACACTCCAGCTGGGAGGCAAGATGCTGGC	62.43	69	1.7
miR-31-5p-reverse	TGGTGTCGTGGAGTCG	55.46		
LIN28B-forward	TTGAGTCAATACGGGTAACAGGA	59.17	184	0.9
LIN28B-reverse	TGACAGTAATGGCACTTCTTTGG	59.18		
GAPDH-forward	ACAGTCAGCCGCATCTTCTT	59.68	94	1.8
GAPDH-reverse	ACGACCAAATCCGTTGACTC	58.21		
U6-forward	CCTCGCTTCGGCAGCACATA	62.91	94	1.3
U6-reverse	ACGCTTCACGAATTTGCGT	59.06		

For RNase R assay, total RNA was exposed to RNase R (Epicentre, Madison, WI, USA) for 15 min at 37 °C. Then the expression levels of circ-FBXW12 and FBXW12 were quantified by RT-qPCR assay.

### Subcellular fraction assay

The PARIS Kit (Invitrogen) was adopted to separate the cytoplasm and nucleus in HMCs according to the manufacturers’ instructions. The level of circ-FBXW12 in the cytoplasm and nucleus was quantified.

### Cell counting kit-8 (CCK-8) assay

To test cell viability, CCK-8 assay kit (Sigma-Aldrich) was used. In short, the transfected HMCs were plated into 96-well plates and then CCK-8 was supplemented into each well for further 2 h of incubation. The absorption was measured at 450 nm via a microplate reader (Bio-Rad, Hercules, CA, USA).

### Enzyme linked immunosorbent assay (ELISA)

The concentrations of interleukin-6 (IL-6) and tumour necrosis factor α (TNF-α) in the culture medium of HMCs were examined by using relevant ELISA kits (ab178013; ab181421; Abcam, Cambridge, MA, USA) referring to the manufacturers’ instructions.

### Flow cytometry analysis

To assess cell cycle process, HMCs with relevant transfection were collected, rinsed with phosphate buffer saline (PBS; Sigma-Aldrich) and fixed for 8 h with 75% ethanol at 4 °C. Then the cells were resuspended in PBS (Sigma-Aldrich) and stained with propidium iodide (PI; Beyotime, Shanghai, China) supplemented with RNase A (Sigma-Aldrich) for 15 min in darkness. Cell proportion in each stage was analyzed through FACScan^®^ flow cytometry (BD Biosciences, San Jose, CA, USA).

### 5-ethynyl-2′-deoxyuridine (EdU) assay

Cell proliferation was assessed by EdU incorporation kit (RiboBio, Guangzhou, China). In short, HMCs were seeded into 24-well plates and EdU was added into the plates for 2 h. Next, the cells were fixed using 4% paraformaldehyde (Sigma-Aldrich) for 0.5 h, permeabilized for 10 min using 0.3% Triton X-100, and rinsed in PBS. Thereafter, the cells were incubated with Aollo fluorescent staining solution for 0.5 h in darkness. Next, the cells were dyed with Hoechst 33342 solution. The positive cells were counted under a fluorescence microscope (Olympus, Tokyo, Japan).

### Western blot assay

The protein was extracted utilizing RIPA buffer (Sigma-Aldrich). Equal amount of proteins was subjected to sodium dodecyl sulfonate-polyacrylamide gel (SDS-PAGE; Sigma-Aldrich) electrophoresis and then blotted on PVDF membranes (Millipore, Billerica, MA, USA). The membranes were then blocked in skim milk, maintained with primary antibodies CyclinD1 (ab226977; Abcam), P21 (ab109520; Abcam), collagen I (ab34710; Abcam), collagen IV (ab6586; Abcam), TGF-β1 (ab215715; Abcam), LIN28B (ab115698; Abcam) and GAPDH (ab37168; Abcam) and secondary antibody (ab205719; Abcam). The immunoblotting signals were visualized using an enhanced chemiluminescence (ECL) kit (Beyotime).

### Measurement of superoxide dismutase (SOD) activity and malondialdehyde (MDA) level

The activity of SOD and the content of MDA in HMCs were examined with SOD assay kit (Sigma-Aldrich) and MDA assay kit (Sigma-Aldrich) strictly according to the guidelines.

### Dual-luciferase reporter assay

The wild-type (including miR-31-5p binding sites) or mutant (miR-31-5p binding sites mutation) fragments of circ-FBXW12 or LIN28B 3’UTR were administrated into psiCHECK-2 plasmid (Promega, Madison, WI, USA). Then HMCs were transfected with the constructed vectors and miR-31-5p/miR-NC. The luciferase intensity was measured with Dual-Luciferase Reporter Assay Kit (Promega) following 48 h of co-transfection.

### RNA immunoprecipitation (RIP) assay

HMCs were lysed in RIP buffer and cell extracts were cultivated with magnetic beads conjugated with IgG (Abcam) or Ago2 (Abcam). Thereafter, the samples were maintained with proteinase K (Sigma-Aldrich) for 0.5 h to separate the RNA–protein complexes from beads followed by RT-qPCR assay for the abundance of circ-FBXW12, miR-31-5p and LIN28B.

### Statistical analysis

The sample size was evaluated by G*Power. The experiments were performed in triple times and the data were estimated by GraphPad Prism 7 and exhibited as mean ± SD. The data were normally distributed. The differences of two sets and three sets were analyzed by Student’s *t*-test or one-way analysis of variance followed by Tukey’s test. It was considered as significant when *P* < 0.05.

## Results

### Circ-FBXW12 was highly expressed in DN patients and HG-induced HMCs

**Fig. 1:**
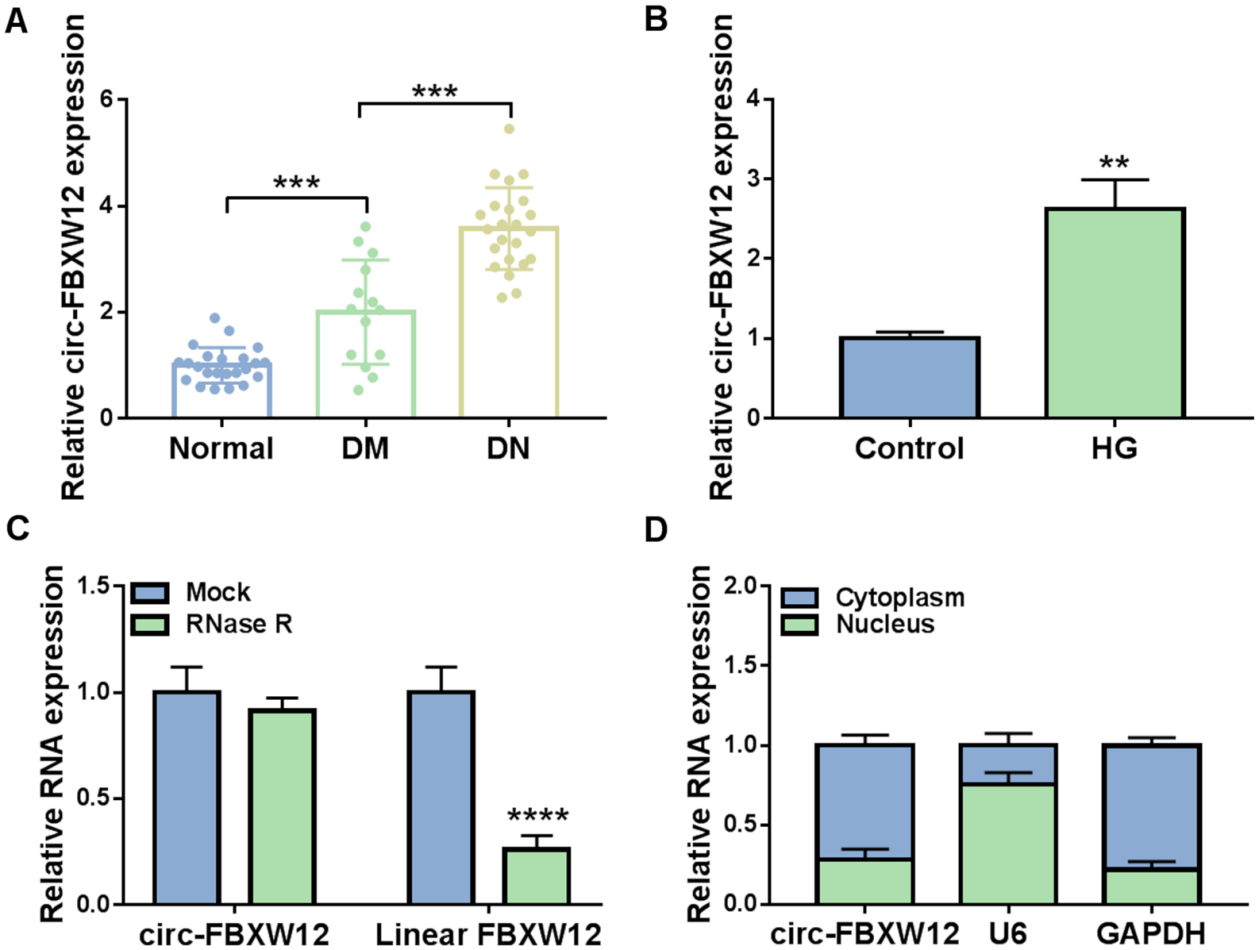
Circ-FBXW12 was increased in DN patients and HG-treated HMCs. **A** The expression of circ-FBXW12 in the serums of DN patients, diabetic patients without DN and normal controls was detected by RT-qPCR assay. **B** The expression of circ-FBXW12 in HG-stimulated HMCs was detected by RT-qPCR assay. **C** The levels of circ-FBXW12 and FBXW12 in HMCs treated with or without RNase R were determined using RT-qPCR assay. **D** The expression of circ-FBXW12 in the cytoplasm and nucleus of HMCs was examined by RT-qPCR assay. **P < 0.01, ****P < 0.0001

To explore the function of circ-FBXW12 in DN progression, the expression of circ-FBXW12 in the serums of DN patients, diabetic patients without DN (DM group) and healthy volunteers was determined by RT-qPCR assay. The results showed that circ-FBXW12 level was apparently increased in DN patients and DM patients in comparison with normal controls (Fig. 1A). Moreover, circ-FBXW12 was markedly increased in HG-treated HMCs compared to control groups (Fig. 1B). RNase R assay indicated that circ-FBXW12 was resistant to RNase R treatment, while linear FBXW12 was digested by RNase R treatment (Fig. 1C). Moreover, our results exhibited that circ-FBXW12 was mainly enriched in the cytoplasm of HMCs (Fig. 1D). These results suggested the potential role of circ-FBXW12 in DN development.

### Silencing of circ-FBXW12 suppressed HG-induced cell proliferation, inflammation, cell cycle, ECM production and oxidative stress in HMCs

**Fig. 2:**
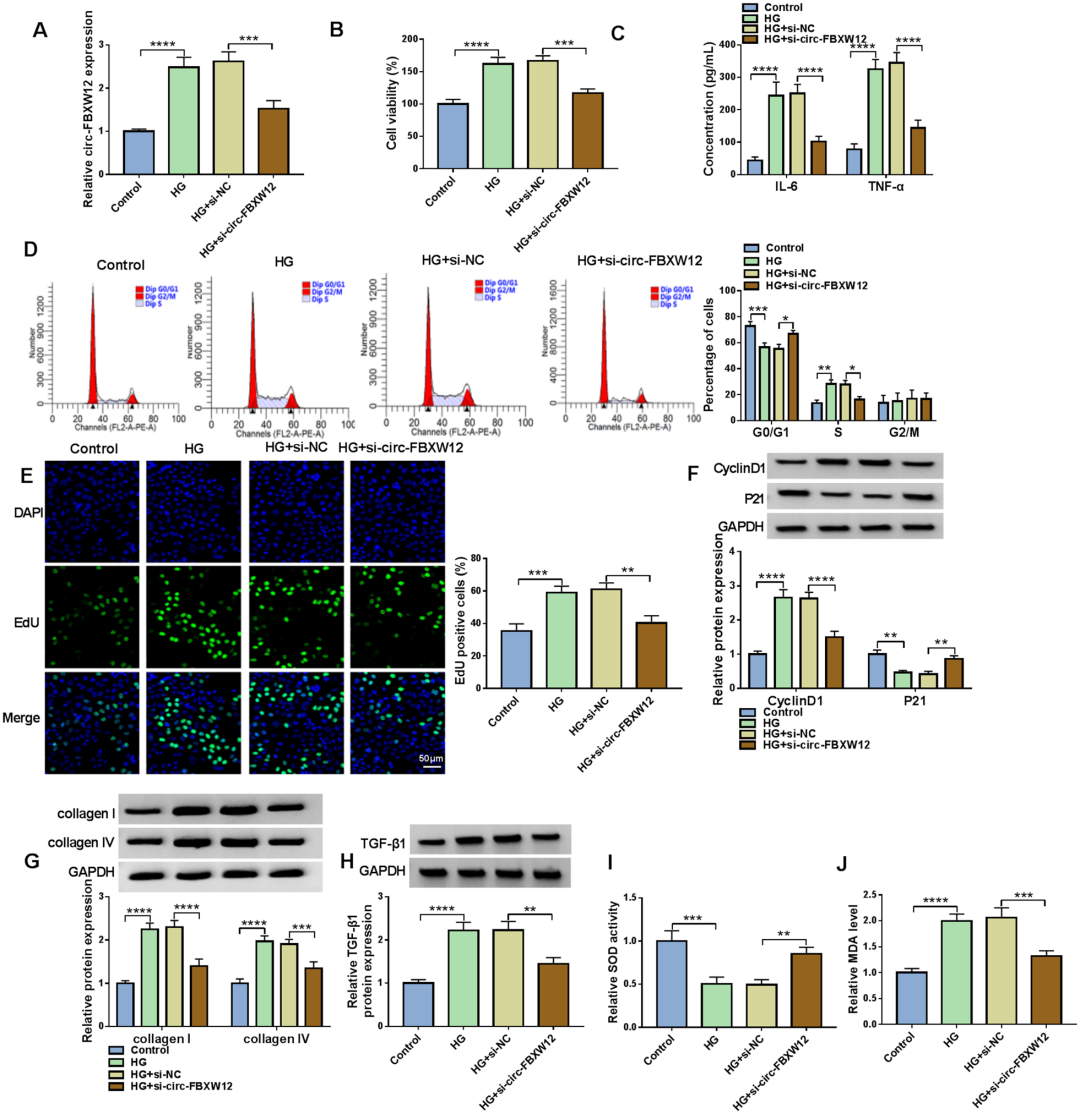
Circ-FBXW12 silencing reversed HG-mediated effects on cell growth, inflammation, cell cycle, ECM production and oxidative stress in HMCs. HMCs were assigned to Control, HG, HG + si-NC and HG + si-circ-FBXW12 groups. **A** The expression of circ-FBXW12 in HMCs was determined by RT-qPCR assay. **B** The viability of HMCs was assessed by CCK-8 assay. **C** The concentrations of IL-6 and TNF-α in HMCs were examined with ELISA kits. **D** The cell cycle process in HMCs was analyzed by flow cytometry analysis. **E** The proliferation of HMCs was evaluated by EdU assay. **F**–**H** The protein levels of CyclinD1, P21, collagen I, collagen IV and TGF-β1 in HMCs were measured via western blot assay. **I** and **J** The activity of SOD and the level of MDA in HMCs were examined with specific commercial kits. **P < 0.01, ***P < 0.001, ****P < 0.0001

To explore the exact roles of circ-FBXW12 in DN development, HMGs were transfected with si-circ-FBXW12 to knock down circ-FBXW12 expression in HMGs. As a result, the upregulation of circ-FBXW12 in HMGs caused by HG treatment was reversed by the transfection of si-circFBXW12 (Fig. 2A). CCK-8 assay indicated that HG treatment led to a distinct promotion in the viability of HMCs compared to control group, while circ-FBXW12 silencing reversed the effect (Fig. 2B). ELISA showed that the concentrations of IL-6 and TNF-α were increased in HMCs treated with HG, whereas the effects were abated by downregulating circ-FBXW12 (Fig. 2C). As illustrated by flow cytometry analysis, the percentage of HMCs in G0/G1 phases was reduced and the percentage of HMCs in S phase was increased after HG exposure, while circ-FBXW12 knockdown ameliorated the effects (Fig. 2D). EdU assay indicated that HG treatment promoted HMC proliferation, with circ-FBXW12 deficiency rescued the impact (Fig. 2E). HG treatment increased CyclinD1 protein level and decreased P21 level in HMCs, with circ-FBXW12 silencing rescued the effects (Fig. 2F). Moreover, we found that HG treatment increased the protein levels of ECM markers (collagen I and collagen IV) in HMCs, while the effects were overturned by decreasing circ-FBXW12 (Fig. 2G). TGF-β1 is closely related to the production of ECM [22]. Thus, we detected the protein level of TGF-β1 in HG-treated HMCs. Our results showed that TGF-β1 was elevated in HG-treated HMCs, but circ-FBXW12 interference reversed the effect (Fig. 2H). Besides, it was found that the activity of SOD was inhibited and the level of MDA was increased in HG-treated HMCs, while circ-FBXW12 silencing rescued the effects (Fig. 2I and J). Taken together, HG treatment promoted cell proliferation, inflammation, cell cycle process, ECM production and oxidative stress in HMCs, with circFBXW12 silencing abrogated the impacts.

### Circ-FBXW12 acted as miR-31-5p sponge

**Fig. 3:**
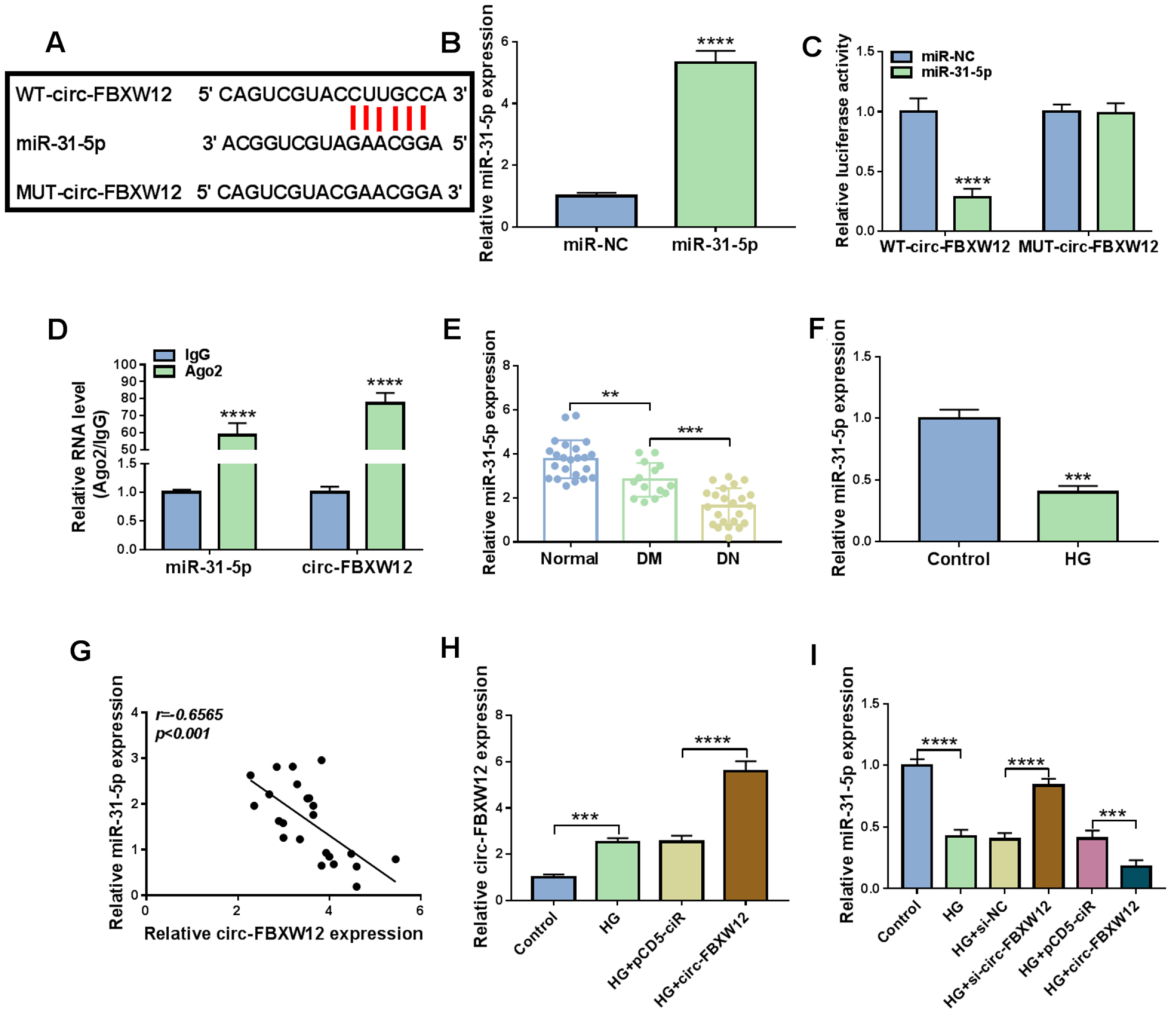
Circ-FBXW12 directly interacted with miR-31-5p. **A** The binding sites between circ-FBXW12 and miR-31-5p. **B** The expression of miR-31-5p in HMCs transfected with miR-NC or miR-31-5p was detected by RT-qPCR assay. **C** and **D** The interaction between miR-31-5p and circ-FBXW12 was demonstrated by dual-luciferase reporter assay and RIP assay. **E** and **F** The expression of miR-31-5p in DM, DN patients’ serums and HG-stimulated HMCs was determined through RT-qPCR assay. **G** The linear correlation between the levels of circ-FBXW12 and miR-31-5p in the serums of DN patients was analyzed by Spearman’s correlation coefficient analysis. **H** The expression of circ-FBXW12 in HMCs transfected with pCD5-ciR or circ-FBXW12 in HG condition was detected by RT-qPCR assay. **I** After HMCs were transfected with si-NC, si-circ-FBXW12, pCD5-ciR or circ-FBXW12 in HG condition, the expression of miR-31-5p was detected by RT-qPCR assay. ***P < 0.001, ****P < 0.0001

By using bioinformatics prediction software circinteractome (https://circinteractome.irp.nia.nih.gov/), miR-31-5p was found to share the binding sites of circ-FBXW12 (Fig. 3A). As exhibited in Fig. 3B, the transfection of miR-31-5p led to a marked elevation in miR-31-5p expression in HMCs. Dual-luciferase reporter assay showed that miR-31-5p overexpression dramatically inhibited the luciferase activity of WT-circ-FBXW12 in HMCs, but did not affect the luciferase activity of MUT-circ-FBXW12 (Fig. 3C). RIP assay showed that the levels of miR-31-5p and circ-FBXW12 were notably enriched in Ago2 RIP groups compared to IgG groups (Fig. 3D). These results indicated the interaction between miR-31-5p and circ-FBXW12. Indeed, miR-31-5p was weakly expressed in DN patients, DM patients and HG-treated HMCs compared to control groups (Fig. 3E and F). As analyzed by Spearman’s correlation coefficient analysis, there was an inverse correlation between the levels of miR-31-5p and circ-FBXW12 in the serums of DN patients (Fig. 3G). Thereafter, we found that HG-induced circ-FBXW12 upregulation was further promoted by the transfection of circ-FBXW12 in HMCs, indicating that circ-FBXW12 was successfully transfected into HMCs (Fig. 3H). Furthermore, our results presented that circ-FBXW12 knockdown increased miR-31-5p expression in HMCs, while circ-FBXW12 overexpression decreased miR-31-5p expression in HMCs (Fig. 3I). Collectively, circ-FBXW12 directly targeted miR-31-5p to negatively alter miR-31-5p expression in HMCs.

### Inhibition of miR-31-5p rescued the impacts of circ-FBXW12 knockdown on cell growth, inflammation, cell cycle, ECM production and oxidative stress in HG-treated HMCs

**Fig. 4:**
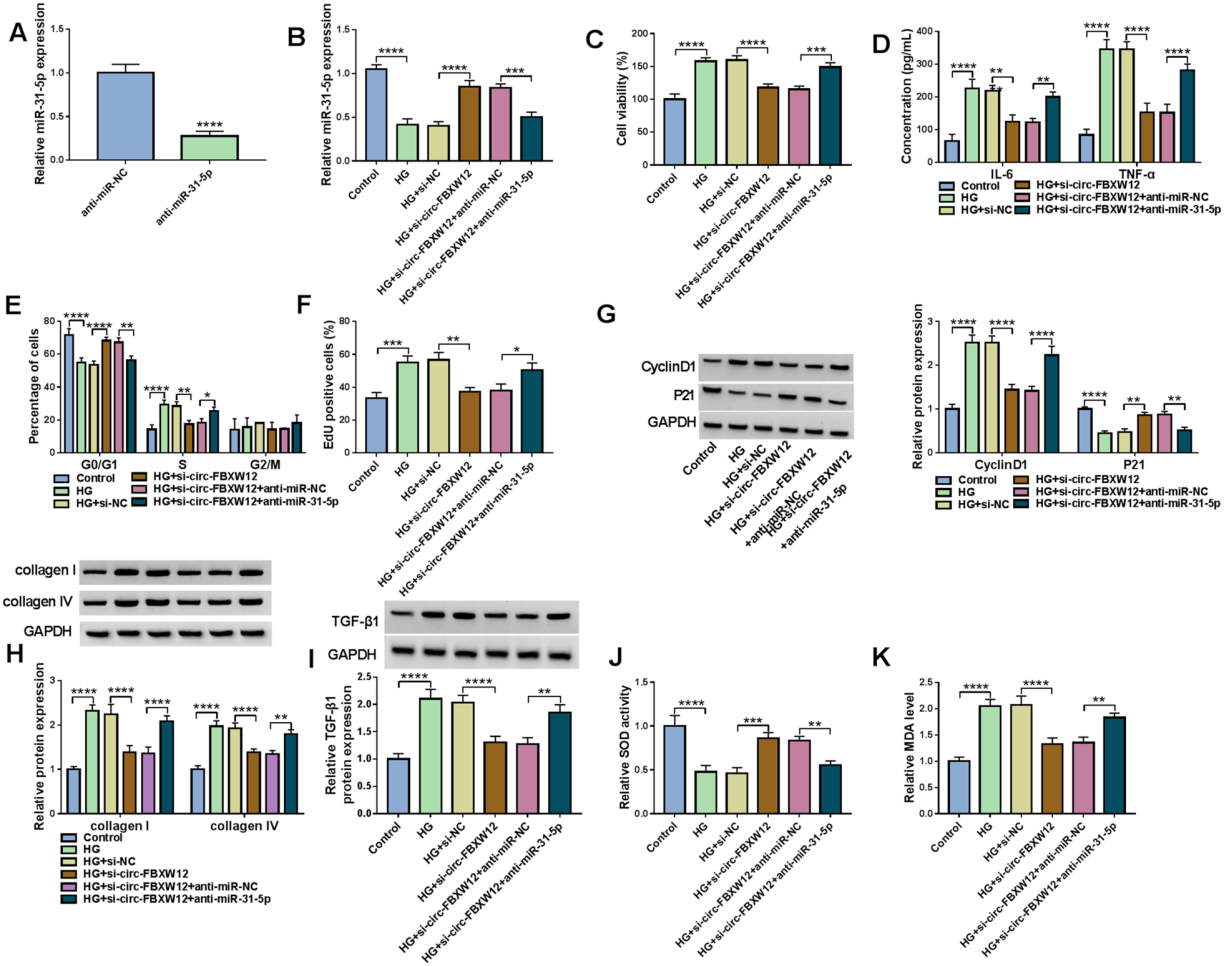
Circ-FBXW12 regulated cell proliferation, inflammation, cell cycle, ECM production and oxidative stress in HG-treated HMCs by targeting miR-31-5p. **A** The expression of miR-31-5p in HMCs transfected with anti-miR-NC or anti-miR-31-5p was determined by RT-qPCR assay. (B-I) HMCs cells were transfected with si-NC, si-circ-FBXW12, si-circ-FBXW12 + anti-miR-NC or si-circ-FBXW12 + anti-miR-31-5p under HG condition. **B** The expression of miR-31-5p in HMCs was detected by RT-qPCR assay. **C** HMC viability was assessed by CCK-8 assay. **D** The concentrations of IL-6 and TNF-α in HMCs were examined with ELISA kits. **E** The cell cycle process in HMCs was analyzed by flow cytometry analysis. **F** The proliferation of HMCs was assessed by EdU assay. **G**–**I** The protein levels of CyclinD1, P21, collagen I, collagen IV and TGF-β1 in HMCs were measured via western blot assay. **J** and **K** SOD activity and MDA level in HMCs were detected with specific kits. *P < 0.05, **P < 0.01, ***P < 0.001, ****P < 0.0001

As we observed in Fig. 4A, anti-miR-31-5p transfection reduced the expression of miR-31-5p in HMCs compared to anti-miR-NC control groups. Next, we explored the relationship of circ-FBXW12 and miR-31-5p in the progression of HG-stimulated HMCs by transfecting si-NC, si-circ-FBXW12, si-circ-FBXW12 + anti-miR-NC or si-circ-FBXW12 + anti-miR-31-5p. It was found that circ-FBXW12 knockdown increased the expression of miR-31-5p in HMCs, while anti-miR-31-5p transfection abolished the effect (Fig. 4B). CCK-8 assay suggested that circ-FBXW12 silencing repressed HG-stimulated HMC viability, with miR-31-5p inhibition reversed the impact (Fig. 4C). ELISA showed that circ-FBXW12 knockdown reduced the concentrations of IL-6 and TNF-α in HG-triggered HMCs, while the effects were weakened by decreasing miR-31-5p (Fig. 4D). As illustrated by flow cytometry analysis, circ-FBXW12 deficiency arrested cell cycle in G0/G1 phase in HG-treated HMCs, whereas miR-31-5p downregulation ameliorated the effect (Fig. 4E). EdU assay indicated the suppressive role of circ-FBXW12 knockdown in HG-treated HMC proliferation was reversed by reducing miR-31-5p (Fig. 4F). We also found that the effects of circ-FBXW12 knockdown on CyclinD1, P21, collagen I, collagen IV and TGF-β1 protein levels in HG-treated HMCs were abrogated by miR-31-5p inhibition (Fig. 4G–I). Besides, circ-FBXW12 silencing increased SOD activity and decreased MDA level in HG-treated HMCs, with miR-31-5p reduction ameliorated the impacts (Fig. 4J and K). To summarize, circ-FBXW12 deficiency repressed cell proliferation, inflammation, cell cycle, ECM production and oxidative stress in HG-treated HMCs by sponging miR-31-5p.

### LIN28B was the target gene of miR-31-5p

**Fig. 5:**
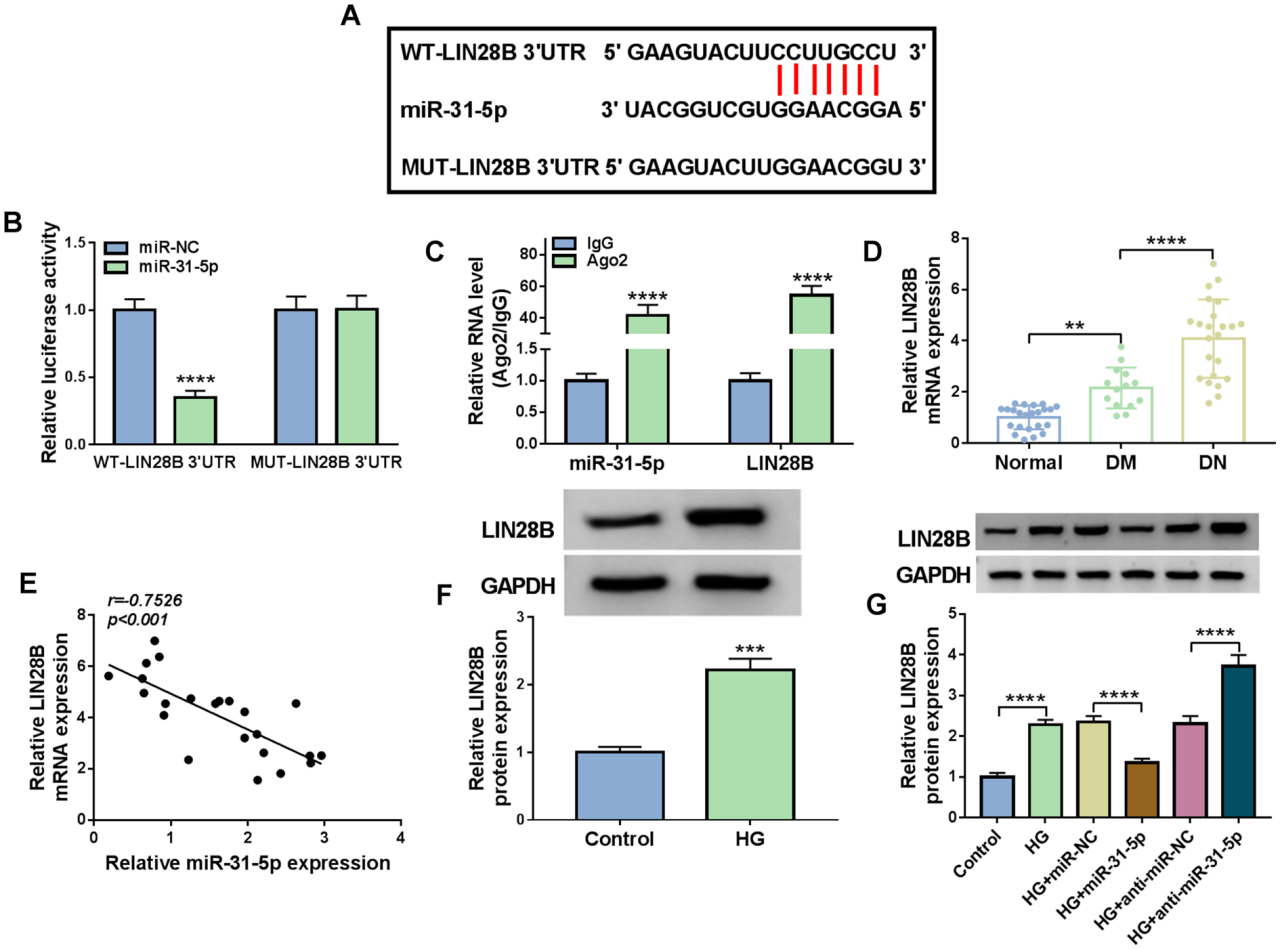
MiR-31-5p directly targeted LIN28B. **A** LIN28B contained miR-31-5p binding sites. **B** and **C** Dual-luciferase reporter assay and RIP assay were conducted to analyze the interaction between miR-31-5p and LIN28B. **D** The mRNA level of LIN28B in the serums of DM, DN patients and healthy volunteers was detected by RT-qPCR assay. **E** The correlation between the levels of mIR-31-5p and LIN28B in DN patients’ serums was analyzed by Spearman’s correlation coefficient analysis. **F** The protein level of LIN28B in HG-treated HMCs was measured via western blot assay. **G** The protein level in HG-treated HMCs transfected with miR-NC, miR-31-5p, anti-miR-NC or anti-miR-31-5p was measured through western blot assay. ***P < 0.001, ****P < 0.0001

To further explore the regulatory mechanism of circ-FBXW12/miR-31-5p in HG-induced HMCs progression, we analyzed starBase v2.0 (http://starbase.sysu.edu.cn/starbase2/) and found that LIN28B might be a target gene of miR-31-5p (Fig. 5A). Then dual-luciferase reporter assay and RIP assay were carried out to demonstrate the prediction. The results of dual-luciferase reporter assay showed that the luciferase activity of WT-LIN28B 3′UTR in HMCs was reduced following miR-31-5p overexpression, while the luciferase activity of MUT-LIN28B 3′UTR was not changed (Fig. 5B). RIP assay indicated that miR-31-5p and LIN28B levels were all enhanced in Ago2 immunoprecipitation complexes compared to IgG control groups (Fig. 5C). Moreover, LIN28B mRNA level in DN patients’ serum was evidently increased and negatively correlated with miR-31-5p level (Fig. 5D and E). Compared to control groups, LIN28B protein level was increased in HG-stimulated HMCs (Fig. 5F). Besides, miR-31-5p overexpression reduced LIN28B protein level and miR-31-5p knockdown elevated LIN28B protein level in HG-treated HMCs (Fig. 5G). These findings illustrated that miR-31-5p negatively regulated LIN28B expression by direct interaction.

### LIN28B knockdown suppressed cell proliferation, inflammation, cell cycle process, ECM production and oxidative stress in HG-treated HMCs

**Fig. 6:**
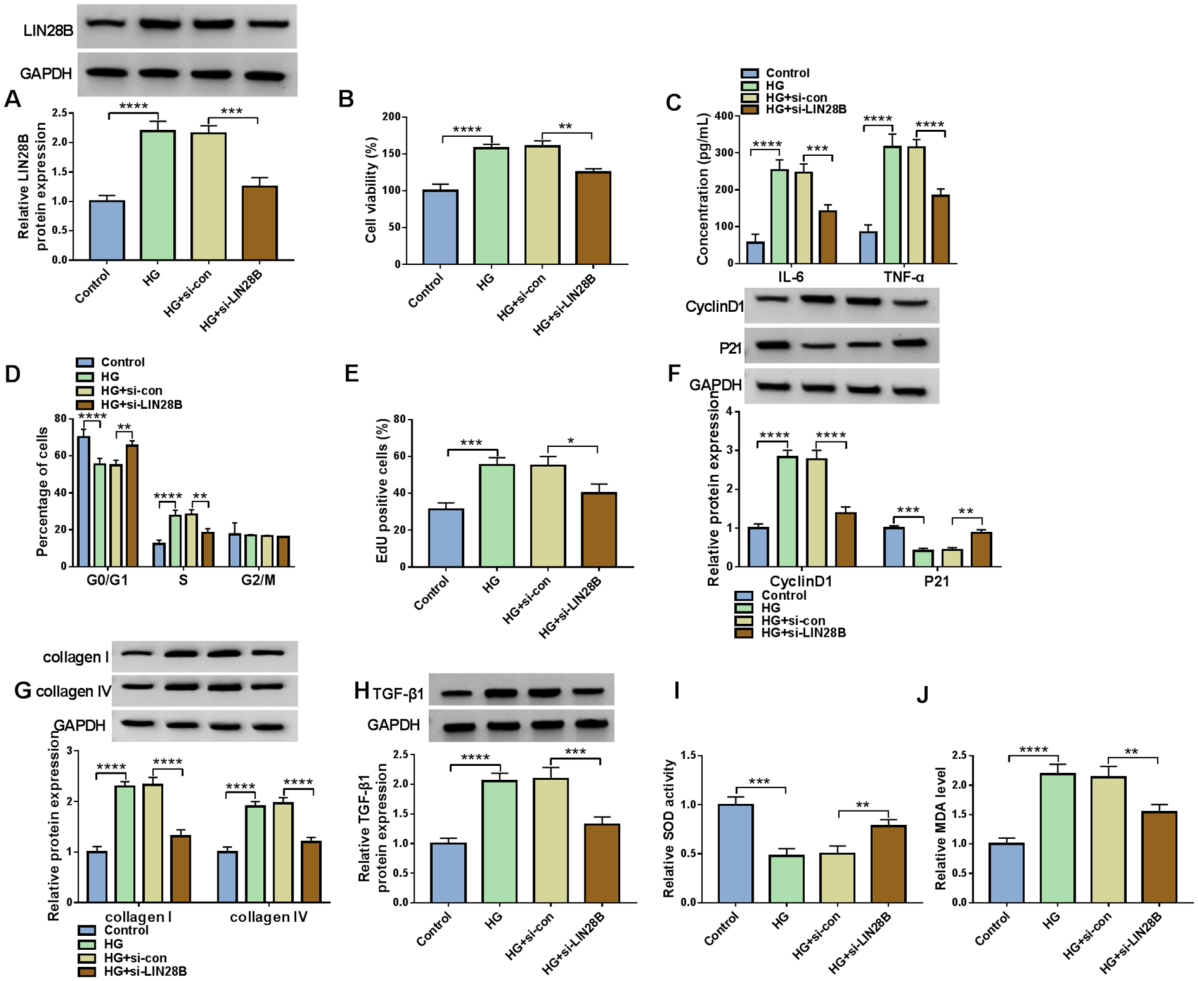
LIN28B knockdown suppressed cell viability, inflammation, cell cycle process, ECM production and oxidative stress in HG-treated HMCs. HMCs were transfected with si-con or si-LIN28B under HG condition. **A** The protein level of LIN28B in HMCs was measured via western blot assay. **B** The viability of HMCs was evaluated by CCK-8 assay. **C** The levels of IL-6 and TNF-α in HMCs were determined by ELISA kits. **D** The cell cycle process in HMCs was analyzed by flow cytometry analysis. **E** The proliferation of HMCs was tested by EdU assay. **F**–**H** The protein levels of CyclinD1, P21, collagen I, collagen IV and TGF-β1 in HMCs were measured via western blot assay. **I** and **J** The activity of SOD and the level of MDA in HMCs were measured by relevant kits. *P < 0.05, **P < 0.01, ***P < 0.001, ****P < 0.0001

Subsequently, the functional roles of LIN28B in HG-induced HMCs progression were investigated. As shown in Fig. 6A, si-LIN28B transfection led to a distinct reduction in LIN28B protein level in HG-treated HMCs. CCK-8 assay showed that LIN28B silencing suppressed HG-induced HMC cell viability compared to si-con control groups (Fig. 6B). HG-induced elevation of IL-6 and TNF-α in HMCs was reversed by silencing LIN28B (Fig. 6C). Flow cytometry analysis showed that the promotional effect of HG treatment on cell cycle process in HMCs was abated by LIN28B knockdown (Fig. 6D). LIN28B silencing inhibited the ability of HG-treated HMCs to proliferate compared to si-con groups (Fig. 6E). Moreover, LIN28B interference reduced the protein levels of CyclinD1, collagen I, collagen IV and TGF-β1 and elevated the protein level of P21 in HG-triggered HMCs (Fig. 6F–H). In addition, we found that LIN28B knockdown enhanced SOD activity and reduced MDA level in HG-stimulated HMCs in comparison with si-con groups (Fig. 6I and J). Collectively, LIN28B knockdown suppressed HG-induced HMCs development.

### MiR-31-5p overexpression repressed cell proliferation, inflammation, cell cycle, ECM production and oxidative stress in HG-stimulated HMCs by targeting LIN28B

**Fig. 7:**
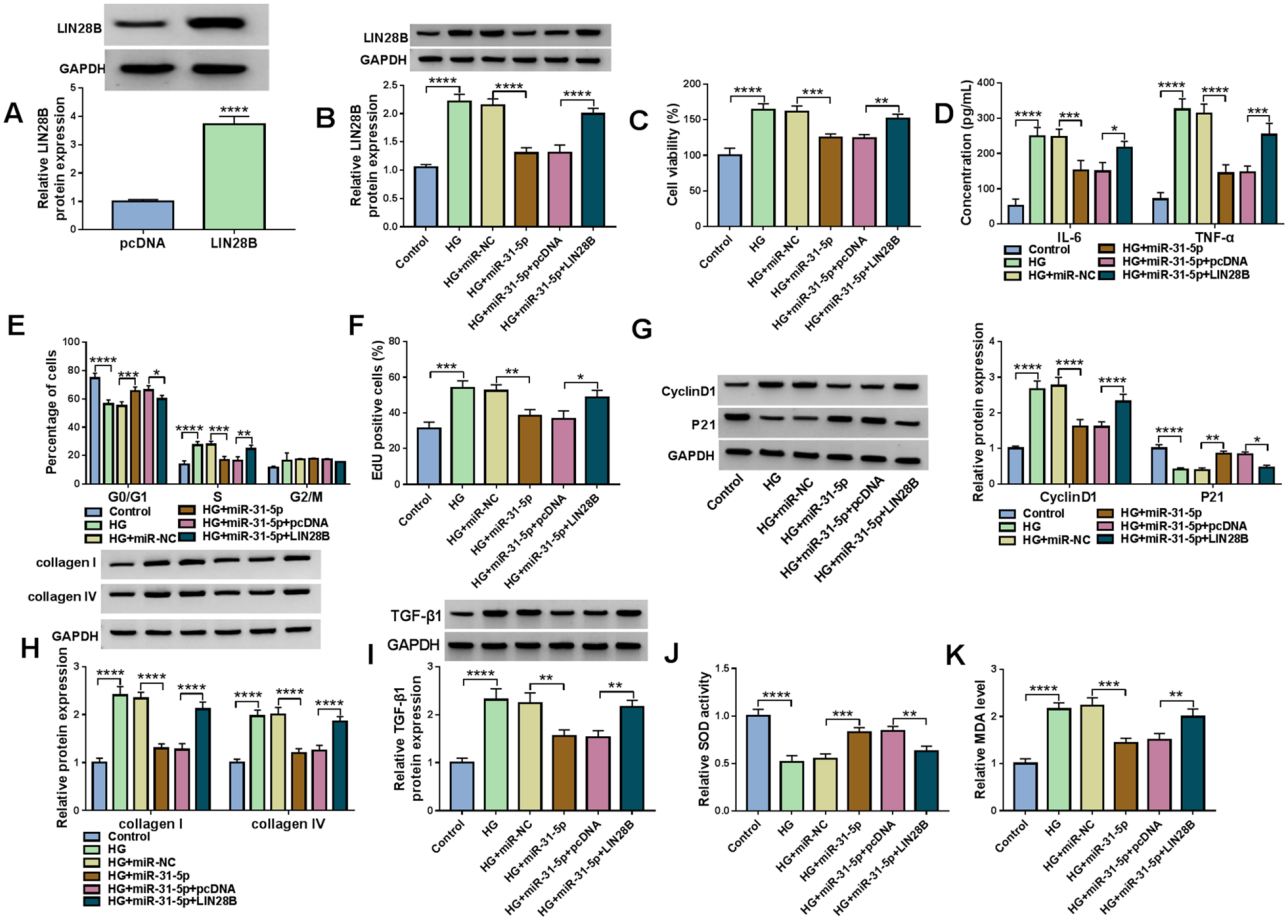
Overexpression of miR-31-5p directly targeted LIN28B to repress the development of HG-stimulated HMCs. **A** The protein level of LIN28B in HMCs transfected with pcDNA or LIN28B was measured via western blot assay. **B**–**I** HMCs were transfected with miR-NC, miR-31-5p, miR-31-5p + pcDNA or miR-31-5p + LIN28B in HG condition. **B** The protein level of LIN28B in HMCs was measured using western blot assay. **C** HMC viability was assessed by CCK-8 assay. **D** The concentrations of IL-6 and TNF-α in HMCs were examined with ELISA kits. **E** Cell cycle in HMCs was analyzed by flow cytometry analysis. **F** HMC proliferation was evaluated by EdU assay. **G**–**I** The protein levels of CyclinD1, P21, collagen I, collagen IV and TGF-β1 in HMCs were measured by western blot assay. **J** and **K** The activity of SOD and the level of MDA in HMCs were examined with commercial kits. *P < 0.05, **P < 0.01, ***P < 0.001, ****P < 0.0001

The transfection of LIN28B overexpression vector elevated LIN28B protein level in HMCs compared to pcDNA control groups (Fig. 7A). MiR-31-5p transfection reduced LIN28B protein level in HG-treated HMCs, while LIN28B transfection reversed the effect (Fig. 7B). CCK-8 assay presented that miR-31-5p overexpression restrained the viability of HG-treated HMCs, while LLIN28B elevation rescued the effect (Fig. 7C). Overexpression of miR-31-5p caused a distinct reduction in IL-6 and TNF-α concentrations in HG-treated HMCs, whereas the impacts were abolished by increasing LIN28B (Fig. 7D). As demonstrated by flow cytometry analysis and EdU assay, miR-31-5p overexpression arrested cell cycle and suppressed cell proliferation in HG-stimulated HMCs, while LIN28B upregulation overturned the impacts (Fig. 7E and F). The protein levels of CyclinD1, collagen I, collagen IV and TGF-β1 were reduced and the protein level of P21 was increased in HG-triggered HMCs after miR-31-5p transfection, while LIN28B overexpression weakened the effects (Fig. 7G–I). Additionally, miR-31-5p overexpression enhanced SOD activity and declined MDA level in HG-treated HMCs, with LIN28B elevation ameliorated the effects (Fig. 7J and K). All these findings suggested that miR-31-5p overexpression suppressed the progression of HG-treated HMCs by targeting LIN28B.

### Circ-FBXW12 knockdown suppressed LIN28B expression by sponging miR-31-5p

**Fig. 8:**
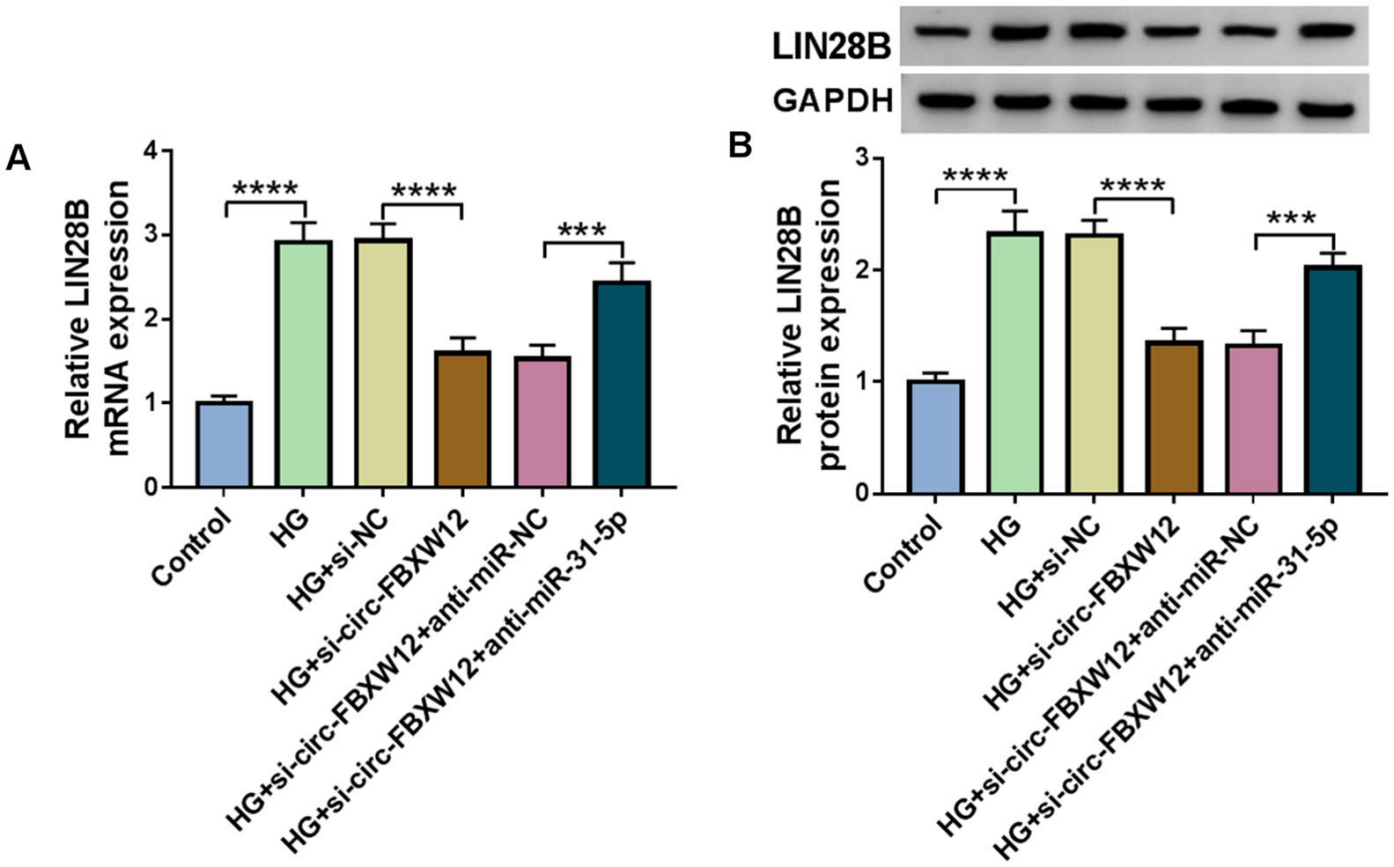
Circ-FBXW12 regulated LIN28B expression by targeting miR-31-5p. **A** and **B** After HMCs were transfected with si-NC, si-circ-FBXW12, si-circ-FBXW12 + anti-miR-NC or si-circ-FBXW12 + anti-miR-31-5p in HG condition, the mRNA and protein levels of LIN28B were determined by RT-qPCR assay and western blot assay, respectively. ***P < 0.001, ****P < 0.0001

At last, the relationships of circ-FBXW12, miR-31-5p and LIN28B were analyzed. It was found that circ-FBXW12 silencing resulted in a marked reduction in LIN28B mRNA and protein levels in HG-treated HMCs, while these effects were rescued by the inhibition of miR-31-5p (Fig. 8A and B).

## Discussion

In the last decade, circRNAs have been gradually identified and discovered to play crucial regulatory roles in a wide range of human diseases [23]. Moreover, the involvement of circRNAs in DN was reported. For example, circ_0000491 level was elevated in DN mice and HG-induced MMCs and promoted ECM production through the mediation of miR-101b and TGFβRI [24]. Circ_0037128 silencing repressed MC growth and fibrosis by reducing AKT Serine/Threonine Kinase 3 (AKT3) through adsorbing miR-17-3p [25]. Circ_WBSCR17 contributed to HG-induced HK2 cell inflammation and fibrosis by miR-185-5p/SRY-box transcription factor 6 (SOX6) axis [26].

In this study, circ-FBXW12 level was abnormally increased in DN patients’ serums. HG treatment also activated circ-FBXW12 expression in HMCs. Thus, we speculated that circ-FBXW12 might exert a function in DN. Proliferation, inflammation, and fibrosis of MCs are important pathophysiological features of DN [27, 28]. Herein, our results showed that HG promoted cell proliferation and inflammatory factors release in HMCs, while circ-FBXW12 knockdown reversed the impacts. In addition, the enhancement of oxidative stress caused by hyperglycemia can aggravate DN progression [29]. In this work, HG-triggered oxidative injury in HMCs was abolished by decreasing circ-FBXW12. As DN is a metabolic disorder characterized by ECM accumulation, which can be induced by some pathological conditions such as oxidative stress and autophagy [30, 31]. Thus, we explored the effect of circ-FBXW12 on ECM by detecting the ECM related markers, including collagen I, collagen IV and TGF-β1 [32, 33]. Our results demonstrated that circ-FBXW12 knockdown reduced the levels of collagen I, collagen IV and TGF-β1 in HG-triggered HMCs, suggesting the suppression of ECM production. All these findings indicated that circ-FBXW12 knockdown was able to alleviate HG-stimulated HMC damage.

Thereafter, the underlying mechanism of circ-FBXW12 was investigated. As a result, we demonstrated that circ-FBXW12 served as the sponge for miR-31-5p and miR-31-5p was lowly expressed in DN patients and HG-triggered HMCs. Though miR-31-5p was reported to be targeted by several circRNAs, such as circ_0035483 [34], circ_0063517 [35] and circ-BPTF [36], the relationship between circ-FBXW12 and miR-31-5p was firstly clarified. Moreover, miR-31-5p inhibition abated circ-FBXW12 knockdown-mediated impact on HG-induced HMC cell injury, indicating that circ-FBXW12 altered HG-induced HMC damage by decoying miR-31-5p. Additionally, our results explored the exact functions of miR-31-5p in HG-induced HMCs. The outcomes exhibited that miR-31-5p overexpression restrained cell proliferation, inflammation, ECM enrichment and oxidative in HG-stimulated HMCs.

Furthermore, it was demonstrated that miR-31-5p directly targeted LIN28B and circ-FBXW12 positively regulated LIN28B expression by sponging miR-31-5p in HMCs. LIN28B silencing ameliorated HG-driven cell growth, inflammatory response, ECM accumulation and oxidative impairment in HMCs. A previous study reported that miR-379-5p could hamper MMC proliferation and ECM production by targeting LIN28B [37]. In this research, LIN28B elevation relieved the influence of miR-31-5p overexpression on HG-induced injury of HMCs.

In conclusion, we elaborated the importance of circ-FBXW12 in HG-induced HMCs. We demonstrated that circ-FBXW12 knockdown could reverse HG-induced HMC cell injury by altering cell growth, inflammation, ECM enrichment and oxidative stress through miR-31-5p/LIN28B axis. Our findings provided clues for understanding the significance of circRNAs in DN and contributed to develop better diagnostic and therapeutic strategies.

However, there are some limitations in our study. For example, the number of patients was limited. Moreover, we did not verify our results in other DN cell model and DN mice model.

## Data Availability

Data analyzed for this study will be available on a reasonable request.

## Abbreviations

DN: Diabetic nephropathy
CircRNAs: Circular RNAs
FBXW12: F-box/WD repeat-containing protein 12
RT-qPCR: Reverse transcription quantitative polymerase chain reaction
LIN28B: Lin-28 homolog B
CCK-8: Cell counting kit-8
EdU: 5-Ethynyl-2′- deoxyuridine
ELISA: Enzyme linked immunosorbent assay
SOD: Superoxide dismutase
MDA: Malondialdehyde
RIP: RNA immunoprecipitation
HG: High glucose
HMCs: Human mesangial cells
ECM: Extracellular matrix
MC: Mesangial cell
ncRNAs: Non-coding RNAs
miRNA: MicroRNA
MMCs: Mouse mesangial cells
MAPK6: Mitogen-activated protein kinase 6
Bach1: BTB Domain And CNC Homolog 1
CCL19: C–C motif chemokine ligand 19
Sema3A: Semaphorin 3A
DMEM: Dulbecco’s modified Eagle’s medium
FBS: Fetal bovine serum
siRNA: Small interfering RNA
GAPDH: Glyceraldehyde 3-phosphate dehydrogenase
IL-6: Interleukin-6
TNF-α: Tumour necrosis factor α
PBS: Phosphate buffer saline
PI: Propidium iodide
RIPA: Radioimmunoprecipitation assay
SDS-PAGE: Sodium dodecyl sulfonate-polyacrylamide gel
PVDF: Polyvinylidene difluoride membranes
ECL: Enhanced chemiluminescence
AKT3: AKT serine/threonine kinase 3
SOX6: SRY-box transcription factor 6
